# The Adoption of AI in Mental Health Care–Perspectives From Mental Health Professionals: Qualitative Descriptive Study

**DOI:** 10.2196/47847

**Published:** 2023-12-07

**Authors:** Melody Zhang, Jillian Scandiffio, Sarah Younus, Tharshini Jeyakumar, Inaara Karsan, Rebecca Charow, Mohammad Salhia, David Wiljer

**Affiliations:** 1 University Health Network Toronto, ON Canada; 2 Institute of Health Policy, Management, and Evaluation, University of Toronto Toronto, ON Canada; 3 Rotman School of Management, University of Toronto Toronto, ON Canada; 4 Department of Medicine University of Toronto Toronto, ON Canada

**Keywords:** artificial intelligence, education, mental health, behavioral health, educators, curriculum

## Abstract

**Background:**

Artificial intelligence (AI) is transforming the mental health care environment. AI tools are increasingly accessed by clients and service users. Mental health professionals must be prepared not only to use AI but also to have conversations about it when delivering care. Despite the potential for AI to enable more efficient and reliable and higher-quality care delivery, there is a persistent gap among mental health professionals in the adoption of AI.

**Objective:**

A needs assessment was conducted among mental health professionals to (1) understand the learning needs of the workforce and their attitudes toward AI and (2) inform the development of AI education curricula and knowledge translation products.

**Methods:**

A qualitative descriptive approach was taken to explore the needs of mental health professionals regarding their adoption of AI through semistructured interviews. To reach maximum variation sampling, mental health professionals (eg, psychiatrists, mental health nurses, educators, scientists, and social workers) in various settings across Ontario (eg, urban and rural, public and private sector, and clinical and research) were recruited.

**Results:**

A total of 20 individuals were recruited. Participants included practitioners (9/20, 45% social workers and 1/20, 5% mental health nurses), educator scientists (5/20, 25% with dual roles as professors/lecturers and researchers), and practitioner scientists (3/20, 15% with dual roles as researchers and psychiatrists and 2/20, 10% with dual roles as researchers and mental health nurses). Four major themes emerged: (1) fostering practice change and building self-efficacy to integrate AI into patient care; (2) promoting system-level change to accelerate the adoption of AI in mental health; (3) addressing the importance of organizational readiness as a catalyst for AI adoption; and (4) ensuring that mental health professionals have the education, knowledge, and skills to harness AI in optimizing patient care.

**Conclusions:**

AI technologies are starting to emerge in mental health care. Although many digital tools, web-based services, and mobile apps are designed using AI algorithms, mental health professionals have generally been slower in the adoption of AI. As indicated by this study’s findings, the implications are 3-fold. At the individual level, digital professionals must see the value in digitally compassionate tools that retain a humanistic approach to care. For mental health professionals, resistance toward AI adoption must be acknowledged through educational initiatives to raise awareness about the relevance, practicality, and benefits of AI. At the organizational level, digital professionals and leaders must collaborate on governance and funding structures to promote employee buy-in. At the societal level, digital and mental health professionals should collaborate in the creation of formal AI training programs specific to mental health to address knowledge gaps. This study promotes the design of relevant and sustainable education programs to support the adoption of AI within the mental health care sphere.

## Introduction

### Background

In the field of mental health, innovations in artificial intelligence (AI) are advancing the ways, in which mental health professionals can diagnose and treat their clients [1]. AI is generally defined as algorithms that enable machines to mimic human reasoning and cognitive functions such as problem-solving, object and word recognition, and decision-making [2]. AI approaches to mental health include machine learning and natural language processing [1]. Machine learning often use algorithms guided by data to predict outcomes and future events [1]. In mental health care, machine learning using data such as those from electronic health records and mobile devices may be used to diagnose various conditions (eg, posttraumatic stress disorder, dementia, and addiction) [1]. Furthermore, AI may be used to diagnose mental health conditions with similar symptoms that require different treatments or subtypes of mental illnesses [3]. Recent developments have been made regarding a classification model for the early and rapid screening of autism spectrum disorder (ASD). This model provides predictions of the disorder at its earliest stages in infancy or early childhood, when individuals have the highest neuroplasticity and there is a greater chance of treating autistic symptoms [4]. Given the time and financial costs of traditional methods of ASD screening [5], this development provides a significant step forward for ASD treatment. When considering the prognosis of mental health issues, using longitudinal data and algorithms can improve the ability of AI to strengthen predictions for patients in psychiatry [1,3]. The use of data to create these predictions has been observed with regard to depression [6], suicide risk [7], and substance abuse [8]. AI can also be used in a variety of methods for mental health treatment. For example, natural language processing can be used to monitor psychotherapy sessions by analyzing the semantic content of each session’s transcripts [9]. This ensures that therapists are conducting interventions that align with their initial specified treatment approach, thus enhancing client-therapist interactions [10]. In addition, AI can be used to predict responses and side effects of mental health treatments (eg, medications, electroconvulsive therapy, and cognitive behavioral therapy) [1]. The discovery of new treatments can also be aided by AI [11], from prevention and diagnosis to treatment delivery and, finally, to support recovery.

The discovery of new approaches to address mental health is essential as increased psychological distress has been observed following the onset of the COVID-19 pandemic [1]. In the United States, for example, 1 in 5 adults reported experiencing mental illness before 2020 [12], with these rates increasing to 2 in 5 adults after social distancing measures were implemented [13]. To appropriately treat mental health issues, early detection and intervention may prove effective in preventing the worsening of symptoms [14]. However, limited access to mental health services has been noted because of several barriers, including a limited number of providers, long wait times, and geographical barriers [15]. With increases in mental illness, it is important for people to access appropriate mental health services in a timely manner [15].

At the same time, there has been a proliferation of digital mobile apps for mental health purposes in which service users and clients can now readily access psychotherapy services and monitor mental health symptoms via their mobile devices [16,17]. This change has been observed alongside the COVID-19 pandemic, which catalyzed the shift toward the digitalization of web-based are for physical and mental health [16,17]. AI-enabled mobile apps were used for diverse purposes, most often for symptom management, consultation (eg, health chatbots), mental health interventions, and socializing with peers [18-21]. Not only were mobile apps conveniently accessible by people during a period of social isolation, but they also offered another potential entry point for vulnerable or underserved populations to seek mental health services. There is also a high level of satisfaction with AI-enabled technologies through the autonomy that clients feel by choosing when, where, and how to seek mental health services [22]. In comparison with being in a provider’s office, some clients may feel more at ease disclosing personal information at home [23].

As AI technologies become more prevalent and clients and service users increasingly opt for digital forms of mental health treatments, professionals in this field must be prepared to not only use AI but also have conversations about it when delivering care [24]. However, despite the potential for AI to enable more efficient and reliable and higher-quality care, there is a persistent knowledge gap among mental health professionals in the adoption of AI [22]. A Swiss study with students in clinical psychology and psychotherapy graduate programs [24] revealed that, although approximately half of the participants expressed interest in learning about AI for mental health care practice, they estimated that education on AI-related topics only contributed to 0.52% of their total degree [24]. Similarly, Kim et al [25] highlighted that, among their sample of millennial medical students and trainees, most reported preferring to use technology in their medical education and practices. However, a lack of structured guidance in the medical education curriculum has created a major barrier to the integration of technologies into real-life clinical contexts [25].

### Objectives

To this end, there is a paucity of established guidelines that could be used to prepare mental health professionals for AI adoption [22]. Although comprehensive frameworks such as AI4People have been developed as guidance to integrate AI into major aspects of society, they do not have a specific focus on mental health care [26]. Therefore, as advancements continue to be made in AI, educational initiatives must be developed for mental health professionals to address the current challenges of AI adoption [27]. There is a need to incorporate and address the needs of mental health professionals to ensure the design and deployment of relevant, effective, and sustainable educational programs. This study aimed to understand current perceptions and attitudes toward AI and the learning needs regarding AI education among mental health professionals. The following research questions were examined:

1. What are the perceptions and attitudes toward implementing AI among mental health professionals?
2. What are the current learning gaps and essential competencies necessary for mental health professionals to effectively integrate AI into their clinical practice?

## Methods

### Study Design

This study was conducted as part of a larger project aimed at accelerating the adoption of AI innovations in clinical care by shifting the mindset, skill set, and tool set of care providers (PRR1-10.2196/30940) [28]. This qualitative descriptive study involved semistructured interviews on the topic of AI in mental health care. Our research is situated within a pragmatism paradigm, which acknowledges that reality is continuously redefined and interpreted according to its usefulness in certain contexts [29]. The research explores some part of reality with the intent of generating knowledge for a deliberate change and improvement [30]. Within this paradigm, we prioritize the practical implications of this work and highlight the importance of using methods that effectively address the research problem [31,32]. Instead of focusing on the conditions and knowledge constructions underpinning the research, pragmatism emphasizes the actions and outcomes of the study [31].

### Recruitment

This study was conducted at the University Health Network, a large academic health science center in Toronto, Ontario, Canada, with recruitment across multiple organizations. The aim of recruitment was to enlist participants working in various professional backgrounds, specialty areas, and locations of practice. To recruit participants, a maximum purposive sampling approach [33] was used whereby researchers identified and selected participants based on their ability to yield relevant information about a particular phenomenon to ensure that the recruited participants were representative of the population of interest. This approach was also undertaken to gain a wide range of perspectives, attitudes, and perceptions [34,35] regarding AI adoption in mental health contexts. Participants were recruited via email invitations sent on behalf of the study team by our partner organizations from the larger study (Canada Health Infoway, Centre for Addiction and Mental Health, Vector Institute, Ontario Association of Social Workers, the Mental Health Commission of Canada, and Associated Medical Services). Individuals were eligible to participate if they identified as mental health professionals in clinical, educational, research, or administrative roles and were able to provide informed consent. Individuals who met the inclusion criteria were invited for a web-based interview at their preferred time.

### Data Collection

Owing to governmental social distancing guidelines during the COVID-19 pandemic, interviews were held on the web via the Teams (Microsoft Corp) videoconference platform. Research assistants with previous experience in qualitative research methods (MZ, SY, and IK) conducted the interviews. A semistructured interview guide consisting of 18 open-ended questions was used to guide the discussions (Multimedia Appendix 1). The questions asked participants to describe (1) their professional background; (2) their perception of and familiarity with digital technology, including AI; (3) their opinions on the relevancy of AI for mental health professions; and (4) potential learning needs about AI. Prompts were included for research assistants to probe the participants when necessary to further explore and understand salient comments and ideas. The interviews were conducted until the researchers felt that no new ideas emerged and data saturation was achieved. Data saturation was defined as a point in the analysis process at which no additional themes emerged from the interviews. During the last few interviews, the participants covered and related similar ideas and concepts. This step was achieved with 20 participants, and thus, no more participants were recruited. All interviews were digitally audio recorded, professionally transcribed, and deidentified. The transcripts and audio recordings were simultaneously reviewed by a research assistant for accuracy.

### Data Analysis

A hybrid approach consisting of both deductive (informed by the interview guide) and inductive (data-driven) analysis processes was used to analyze the data. This approach allowed for the initial analysis of the data using predefined constructs followed by an inductive analysis to identify new themes. Data were analyzed for emerging themes using NVivo (version 12; QSR International), a qualitative data analysis software. In line with the systematic process outlined by Braun and Clarke [36], the interview transcripts were first read by the research assistants to ensure familiarization with the data. In the second step, initial codes were generated. In total, 2 research assistants (MZ and SY) independently analyzed the first 4 transcripts through an exploratory lens and developed an initial coding structure. Subsequently, the remaining transcripts were analyzed by research assistants on the study team (IK, TJ, and JS), with 2 research assistants coding each transcript. The transcripts were analyzed line by line. The codes were then organized into thematic categories and discussed with the larger study team before finalizing the themes. Themes were identified when multiple participants made similar statements or repeated ideas. Segment and document memoing were performed throughout the analytic process to keep track of the initial ideas and understand the thought processes for why certain approaches were taken. This was beneficial when synthesizing the data into higher levels of analytical meaning. Coding comparison queries were run on NVivo to ensure a high percentage of intercoder agreement and for the rigor of the data. Iterative discussions with the research team helped contextualize the overarching themes and resolve disagreements. The research team consistently engaged in reflexivity and reflected on the beliefs, values, and experiences they brought to the study.

### Ethical Considerations

Email invitations were sent to potential participants along with a consent letter outlining further details about the study, the Teams privacy policy, and data handling. The voluntary nature of the study was reiterated at the beginning of the interview, and verbal informed consent was obtained. Participants were informed that the interviews would be recorded for analysis purposes only and would only be accessible to the study team. In addition, participants were advised that they would be able to withdraw consent at any point during the interview. Each consented participant was assigned a study identification number so that their anonymity could be protected throughout the study. Interview transcripts were deidentified before data analysis. All the information gathered by the study team was stored in confidential databases on secured University Health Network servers. This study was approved by the University Health Network Research Ethics Board (ID: 20-6148.4).

## Results

### Overview

A total of 20 interviews were conducted, of which 11 (55%) were conducted with female participants and 9 (45%) were conducted with male participants. Participants were 50% (10/20) mental health practitioners (eg, mental health nurses and social workers), 25% (5/20) educator scientists (both professors or lecturers and researchers), and 25% (5/20) practitioner scientists (3/5, 60% with a dual role as researchers and psychiatrists and 2/5, 40% with a dual role as researchers and mental health nurses). Most participants (18/20, 90%) were from Ontario, representing both large and small urban cities. In total, 10% (2/20) of the participants were located outside Ontario. These locations are not specified to protect the participants’ identity. Participant demographics are described in Table 1.

The duration of the interviews ranged from 19 to 34 minutes. On average, although participants in the categories of *educator-scientist* or *clinician-scientist* reported being comfortable with digital technology and having at least a minimal level of understanding of AI, those in the category of “*mental health practitioner*” (eg, social worker and mental health nurse) reported moderate comfort using digital technology and having little to no knowledge of AI. Thematic analysis of the data yielded four major themes, each with its respective subthemes: (1) fostering practice change and building self-efficacy to integrate AI into patient care; (2) promoting system-level change to accelerate the adoption of AI in mental health; (3) addressing the importance of organizational readiness as a catalyst for AI adoption; and (4) ensuring that mental health professionals have the education, knowledge, and skills to harness AI in optimizing patient care (Textbox 1).

**Table 1:** Characteristics of those who took part in the semistructured interviews (N=20).

Characteristic				Participants, n (%)
**Demographics**				
	**Professional background**			
		Educator scientist		5 (25)
		Practitioner scientist		5 (25)
		Social worker		9 (45)
		Mental health nurse		1 (5)
	**Sex**			
		Male		9 (45)
		Female		11 (55)
	**Location setting**			
		Urban		15 (75)
		Small urban		5 (25)
	**Location**			
		Ontario, Canada		18 (90)
		Outside Ontario, Canada		2 (10)
**AI^a^ knowledge**				
	**AI information source**			
			Family and friends	5 (25)
			Career	8 (40)
			Scholarly articles	3 (15)
			Social media	3 (15)
	**Familiarity with AI**			
			Not at all familiar	8 (40)
			Somewhat familiar	10 (50)
			Very familiar	2 (10)

^a^AI: artificial intelligence.

**Summary of key themes and subthemes derived from the thematic analysis of the interviews.**

**Fostering practice change and building self-efficacy to integrate artificial intelligence (AI) into patient care**

- Establishing awareness and building capacity to engage in conversations about AI and its applicability to mental health
- Bridging the gap between AI and a humanistic approach to mental health care through a mindset shift
- Addressing implementation challenges and fear of AI is needed to promote AI

**Promoting system-level change to accelerate the adoption of AI in mental health**

- Funding constraints in mental health care limit AI prioritization
- Limited time and competing clinical priorities affect AI adoption
- Addressing concerns with regard to bias in data sets and accessibility of AI is crucial in overcoming resistance

**Addressing the importance of organizational readiness as a key catalyst for AI adoption**

- Enhancing transparency and trust through standardization and validation of data and AI tools
- Cultivating trust and managing change to promote the adoption of AI

**Ensuring that mental health professionals have the education, knowledge, and skills to harness AI in optimizing patient care**

- Understanding the impact of AI on quality of care
- Developing a competency-based AI curriculum for mental health professionals
- Incorporating a mentorship component in AI education curricula

### Theme 1: Fostering Practice Change and Building Self-Efficacy to Integrate AI Into Patient Care

#### Subtheme 1: Establishing Awareness and Building Capacity to Engage in Conversations About AI and Its Applicability to Mental Health

Mental health professionals reported varying levels of awareness of technology use in their work settings. Several participants reported a lack of understanding of what the term *AI* referred to and noted that it was common among mental health providers to not have previous training in AI. This extended to a lack of understanding of the associated strengths and limitations of AI. One participant noted the importance of gaining knowledge outside their working environment as their organization may not introduce them to this technology:

I think the general level of awareness, when people start hearing about this, really enables them to see above and beyond their primary focus and their primary locus of wherever they exist, because we’ve become kind of siloed, where you work is what you see. And then if you work there for a long period of time, and you don’t do other things on the outside...you get used to “this is the only things that exist.”

ID 18

In addition, some participants expressed an interest in learning the research findings associated with AI in mental health as they felt that it could increase their understanding of why these technologies are being adopted and how they can be used.

#### Subtheme 2: A Mindset Shift Is Needed to Bridge the Gap Between AI and a Humanistic Approach to Mental Health Care

Participants noted uncertainty as to how the traditional humanistic approach to mental health care may be conducted in conjunction with AI use in mental health. Participants mentioned that their training emphasized understanding the nuances of each client and considering the full person in care. They expressed concern that AI may not be able to recognize these aspects and, instead, base its decisions on previous data. Furthermore, some AI tools were thought of as a “one-size fits all” approach that does not consider each client as a unique case:

The tool is standardized. It doesn't take into consideration all the little nuances of the clinical environment and each client and each staff [and] what they bring into this discussion.

ID 3

Furthermore, participants stressed the importance of a strong relationship with clients when delivering care. Mental health professionals highlighted the importance of being able to connect with clients during therapy, which enables them to monitor the progress of their clients and understand when they may need additional care. Person-to-person interaction was identified as a crucial element of care by professionals, particularly in the mental health field as clients may be in a vulnerable state and less willing to share personal information without a trusting bond. With a trained skill set to form a therapeutic bond, one participant suggested that mental health professionals are slower to adopt AI in general because of the fear that AI could “interfere with or undermine the interpersonal interaction and the [client-therapist] relationship” (ID 8). Some participants also expressed concern for their clients, who may feel uncomfortable interacting with AI. Given their previous training and the engrained values of humanistic care, education is crucial to address mental health professionals’ concerns about losing the human-to-human connection once AI is used.

#### Subtheme 3: Addressing Implementation Challenges and Fear of AI Is Needed to Promote AI

A lack of awareness of the role of AI was linked to resistance and negativity toward implementation. When asked about their initial thoughts on AI, some participants expressed fear. For example, one participant (ID 10) noted that, when it comes to AI, “the first word that comes to mind is horror.” This may be due to a lack of knowledge of AI as that participant (ID 10) expanded that they “know that [they are] probably quite naïve” and were unsure of the types of AI used in practice. Furthermore, several participants reported a lack of understanding of AI in mental health care, thus fearing that it may replace their role as providers. In addition, participants noted concerns that an increased burden would be placed on the professional as they would be responsible for learning about the technology in addition to their current duties. This was especially true among older professionals, who may be less comfortable with technology. One participant stated that “for older people, they’d be a little intimidated” (ID 14), which may lead to hesitancy to incorporate AI into practice.

Participants also feared a decrease in the quality of care with the integration of AI. Participants noted that mental health care is often reliant on forming strong connections between clients and professionals and creating a safe environment. Participants expressed a fear of decreased connection when technology is incorporated into care. For example, there was a decreased ability to read the nonverbal signals of clients when clients and clinicians did not conduct in-person sessions. This fear of AI adoption has also been observed in clients. One participant noted that clients might have concerns about technology being involved in their care. Therefore, it is important that professionals have the skills not only to use these tools but also to explain their use and importance to clients:

I think in our sector, the main thing is how will clients respond to that? Or what will be the impact on clients? And how will they respond?...in that sense, I think that’s always the fear that I hear people have, like, “Oh, are we going to be working with robots now?” So, I think in this sector, that’s a heightened concern because we work in such a human connection, attachment-oriented way that those would just be the considerations on my mind and the biggest challenge would be how people would feel about it.

ID 15

### Theme 2: Promoting System-Level Change to Accelerate the Adoption of AI in Mental Health

Participants touched on contextual factors outside of personal perceptions that can serve as barriers to the adoption and implementation of AI in mental health workplaces. Funding constraints, competing work priorities, and biases in AI algorithms were all commonly described barriers to mental health.

#### Subtheme 1: Funding Constraints in Mental Health Care Limit AI Prioritization

A lack of funding in the field of mental health was frequently expressed by participants. Participants perceived mental health disciplines to be severely underfunded, with certain organizations struggling to provide even the minimum level of care. As one participant (ID 2) mentioned, in comparison with other medical specialties such as “oncology, cardiology, and pediatrics...mental health is often like the poor cousin of other clinical areas.” The participant further noted that, for AI adoption, “in terms of research, funding and beyond...we just don’t have funds and resources to have made any significant advances in the space.”

Funding may also vary depending on the practice setting. Different settings may pose a challenge to standardized training, and as a participant (ID 7) mentioned, “it’s hard to train because, how do you reach out to a lot of these community centers, who are smaller treatment facilities, who don’t have enough resources to even do what they’re doing today?” Mental health providers working in self-funded private practices expressed similar sentiments. In comparison with larger health care organizations, private practices face funding restraints that limit the ability to purchase technology or engage in training:

If clinicians are training to just wanting to be able to have their own little private practice...they may not have the funds to support an adoption of that type of system. They may just forego it altogether, and not really pay attention to it.

ID 13

Overall, limited funding was perceived as a significant challenge in AI adoption. Participants emphasized that, for large-scale changes in AI adoption in mental health to occur, organizational leaders must prioritize funding in research, development, and personnel costs.

#### Subtheme 2: Limited Time and Competing Clinical Priorities Affect AI Adoption

Competing clinical priorities can pose a challenge for the adoption of AI in mental health. With limited time, mental health professionals may opt out of AI training or educational activities, as one participant stated:

If I had to choose between clinical training for some risk assessments, versus for AI, I’ll probably go for the former. Because that is directly impacting my work, and I can see it.

ID 3

Furthermore, training to adopt AI in mental health care practice may require a significant amount of effort. Background requirements for AI such as mathematics and informatics knowledge may not be prevalent among mental health professionals. As such, mental health professionals may perceive that there is too much effort needed to engage in training. One participant (ID 12) mentioned that mental health providers “don’t necessarily have the time to take another course from the start and go back to learning new concepts...they are already overwhelmed with clinic practice.” Hence, heavy workloads and competing priorities in the mental health discipline create difficulties in implementing new technologies into the workflow.

#### Subtheme 3: Addressing Concerns With Regard to Bias in Data Sets and Accessibility of AI Is Crucial in Overcoming Resistance

Mental health professionals are concerned with the appropriateness of AI data sets. Given that mental health professionals work with clients and service users who may come from racialized or marginalized populations, issues such as biases in AI algorithms can be alarming. For instance, a participant expressed their hesitancy toward the representativeness of AI technologies:

We handle a number of racialized folks that we care for. I think we hear a lot about is biases, particularly as we think about certain groups, like racialized groups and potentially not having a representativeness in some of the datasets in which we generate some of these learning...so the last thing we’d want to do is be applying learning from AI, that was not appropriate, because the dataset it was generated on didn’t include these folks. So, you know, I think that’s a bit of a hesitation when it comes to the mental health context.

ID 2

Accessibility concerns were also prevalent among participants. As described by a participant (ID 13), “as a social worker, you deal with a lot of patients and clients that come from low socioeconomic statuses and just don’t have access to the type of things that would facilitate access to AI.” Furthermore, another participant (ID 8) mentioned that individuals with cognitive disorders may have difficulty accessing or navigating digital technology platforms. The participant elaborated further that these individuals encounter difficulty with videoconferencing platforms such as Teams. Thus, clients with mental illnesses, including cognitive disorders, may experience restricted access to technologies given their “limited ability to learn new things” (ID 8).

In general, participants acknowledged the growing acceptance of AI. Despite this, concerns regarding equity, diversity, and inclusivity remain. Without addressing these issues, resistance will persist in a large population of mental health professionals.

### Theme 3: Addressing the Importance of Organizational Readiness as a Key Catalyst for AI Adoption

Organizations play a significant role in the adoption and implementation of AI technologies. Shifts in the current workflow need to start at the organizational level to create greater employee buy-in and trust and minimize disruption.

#### Subtheme 1: Standardization and Validation of Data and AI Tools to Enhance Transparency and Trust

Having standardized AI systems increases user confidence. Participants mentioned that it would be ideal for their organizations to implement federally approved and validated AI tools. This way, professionals are assured of the standard and quality of the tools that they integrate into their care. However, there are no existing accreditation programs in Canada. As such, standardized guidelines to safely implement AI in health care organizations are lacking. In addition to large academic health care networks, smaller-scale hospitals do not have the resources to conduct “quality evaluations [that will] validate the tools they choose and the way they implement them” (ID 7). Even within larger institutions, the absence of validated implementation frameworks can lead to lower confidence in using AI technology. As mentioned by one participant, the generalization of health care diagnosis using AI algorithms is quite disappointing, such that “if you take another data set from another hospital, or another country, even, the ‘world can collapse.’” Furthermore, although there are some positive cases of AI, this is not observed “in mental health and psychiatry, [where it is] still really, really disappointing” (ID 12). Participants expressed the need for federal-level standards of evaluation to “provide guidance on quality assurance” (ID 7). Proper infrastructure to develop, implement, and validate AI is crucial for health care organizations to establish the trust and confidence of their providers.

#### Subtheme 2: Cultivating Trust and Managing Change to Promote the Adoption of AI

Participants noted that, before pushing employees to adopt new technology into their work routine, there first need to be changes within the overall organization. As one participant (ID 18) mentioned, in the advent of technology implementation, “there are existing workflows where it requires some change management, culturally, to get people to buy into this idea, demonstrate that it can be useful and that it can result in patient benefits.” Employees seek clarity on innovative tools and a strong rationale for adopting them. Organizations must engage in socialization to communicate the value and risk and demonstrable benefits of AI for health care professional buy-in. Thus, employee buy-in may not happen unless the change occurs in the organization’s overall implementation approach:

One is, well, how do we communicate value to them? Everyone has their own disposition towards AI, what it can do, and the risks and their tolerance for risk. So, I think understanding that is key to getting clinicians to onboard or having clinician buy-in.

ID 19

Important information about AI that participants would like to receive from their organizations includes current research conducted on AI technologies. One participant (ID 5) expressed that being aware of recent research findings could enable them to feel informed and empowered to make decisions about their use of AI. They noted that, in comparison with their colleagues, their exposure to research and understanding of the evolving field of AI made them “more aware and open to adopting technologies into their practice.” Another participant further argued that, as professionals that constantly provide care to people, being informed is crucial to engage in conversations about AI and play a part in the decision-making process.

### Theme 4: Ensuring That Mental Health Professionals Have the Education, Knowledge, and Skills to Harness AI in Optimizing Patient Care

#### Subtheme 1: Understanding the Impact of AI on the Quality of Care

As noted previously, participants with a lack of awareness of AI expressed fear that technology might decrease the quality of care. Education of mental health professionals to increase knowledge of the role of AI in care is important for its uptake. This can be accomplished by ensuring that professionals have a basic knowledge of and familiarity with AI. Although some professionals may require extensive learning, a deeper knowledge of AI (eg, algorithm development) is not required of all professionals. Instead, a basic understanding of the development, evaluation, and outcomes of these tools would be beneficial:

Another challenge is the education. And at the big pedagogic level, being able to have clinicians in mental health that are knowledgeable, not necessarily about the details, but having a broad perspective about what AI can do, what AI can’t do, what are the intrinsic biases, etc. And so that’s why I designed with my coding [inaudible]course, about computational medicine to try to raise this new generation of clinicians that is going to be familiar with core concepts of AI machinery and computational methods.

ID 12

Some participants stated that they would like to learn more about how these tools are optimizing their care as there is a lack of awareness of why they are being implemented. One participant noted that they were interested in “understanding the purposes of artificial intelligence in health care...[and] know more about the impact it’s having in improving the quality of care” (ID 3) for both the client and the provider. Understanding how the tool may reduce participants’ burden may also lead to increased comfort and likelihood of use. Furthermore, training could increase understanding in mental health professionals about the safety and risk of integrating tools into care:

I would want to have an opportunity to use it with a seasoned practitioner and understand exactly what it’s doing. Especially where we’re talking about our brain, I would want to know for sure that it couldn’t, or wouldn’t, cause any harm to the client...I would want to really understand how it could be helpful in terms of the outcomes.

ID 11

Nevertheless, there were varying levels of knowledge among participants as to how to access this education. Although some participants reported that they had previously participated in AI workshops or courses, others lacked experience but expressed that they were interested in this type of education and would be willing to participate in future programs or events.

#### Subtheme 2: Developing a Competency-Based AI Curriculum for Mental Health Professionals

Participants expressed a desire for the education of mental health professionals on the role of AI in practice and perceived current AI training opportunities for mental health professionals as lacking. In particular, a participant noted that the practicality of AI has not “been so clearly communicated to frontline clinicians” (ID 20). Although there is emerging evidence showing the practical use of AI, such as predictive modeling in social media use to predict the risk of suicide, “the real challenge remains as to what the frontline clinician can practically use.” Given that minimal training or education opportunities are being offered, mental health professionals “cannot actually see how AI is translated to their individual practice” (ID 20) and, thus, do not consider AI as a priority. As such, participants feel that, if their organizations are to bring AI approaches into decision support, they must “feel empowered and supported in using it, through training” (ID 2). As a start, organizations should advocate for the importance of AI training by offering a curriculum that would support the safe implementation and adoption of AI tools.

Including AI in mental health education curricula can assist mental health professionals’ understanding and awareness of technology in the field. Participants noted that the current curriculum does not include this information, and therefore, a knowledge gap exists among professionals. Some participants suggested that this education should take place before entering the workforce and be included in undergraduate and graduate education curricula. Other participants suggested that this education could take place through continuing professional development courses:

I have to say that if, as far as education goes, there is a lot of work that can be done in schools and universities. Not just in health care, but also in schools because a lot of students in schools are going to need mental health support. So, it’s not just as a society, different institutions need to educate, like, not because people are not smart or anything, but they need to be educated in this area, as well.

ID 5

Some participants noted that creating a standard, mandatory course for mental health students may be difficult because of the limited number of AI experts in the mental health field who could take on the role of instructors. This paucity of expert instructors may constrain the number of available courses for mental health professionals:

Having education on it would be beneficial. The only issue I find that I could see coming up with that would be...finding people [who] are able [to be] experts essentially to be able to speak on that and teach.

ID 13

In addition to being made aware of the broad scope of AI in health care, mental health professionals must be trained on how to use these tools to incorporate them into their practice. This training is contingent on having a team that can support the education of professionals and monitor the use of the tool over time:

If you’re going to authorize an app to be used across the province, hospitals have no idea of how to do this...so I think first we’re going to have to figure out how to do it safely, how to implement it, and then we’re going to have to train people on how to do that. So, I think that’s sort of a curriculum that needs to be developed.

ID 7

#### Subtheme 3: Incorporating a Mentorship Component in AI Education Curricula

Consistent support in the education on AI is important. The format of a short didactic session was noted as insufficient for understanding how to use a tool and ensuring its uptake in everyday use. Having access to an AI expert or mentor who could answer questions, engage with users, and advocate for the specific technology’s use was seen as beneficial in learning how to use these tools. It is also important to involve mentors who have previous experience implementing AI when educating professionals. Providing this mentorship gives the ability to understand and respond to each user’s unique needs:

“So, I guess training, of course, will be one, some support for troubleshooting because you do training and you start using it. If you can go back to somebody to troubleshoot as you are using it in more different, diverse situations, or you are stuck and you don’t know what to do about it. So, having that resource person, or at least support systems somewhere. Then some kind of community of practice or something, maybe within the organization or outside organization to explore diverse ways of using that technology, learning from others, their experiences as well and sharing your own challenges.”

ID 3

As discussed previously, the limited number of experts in the mental health care AI field may make it difficult to identify appropriate mentors. Therefore, it is crucial to enhance the skill set of those in the field to produce future experts who can assist in a mentorship capacity.

## Discussion

### Principal Findings

Advances in digital technologies such as AI in the field of mental health care have increased significantly in recent years, and it is vital to understand the role that mental health professionals play in AI adoption. For these tools to be adopted, it is essential that mental health professionals have the knowledge and skills required for their use. Our analysis identified 4 themes: fostering practice change and building self-efficacy to integrate AI into patient care; promoting system-level change to accelerate the adoption of AI in mental health; addressing the importance of organizational readiness as a catalyst for AI adoption; and ensuring that mental health professionals have the education, knowledge, and skills to harness AI in optimizing patient care. These findings are supported by previous literature. For example, our finding that negative or fearful perceptions of AI may be due to minimal exposure to it is in line with the findings of Blease et al [37], who discovered that differing levels of enthusiasm and fear in using AI may result from a lack of knowledge of it. Furthermore, our finding that older adults may be uncomfortable with AI integration because of low digital literacy was also observed in one article that noted the importance of tailoring digital mental health interventions aimed at older adults [38]. Other studies have also highlighted a lack of standardized guidelines on development, clinical integration, and training to adopt and use AI in mental health services [39-42].

**Figure 1:**
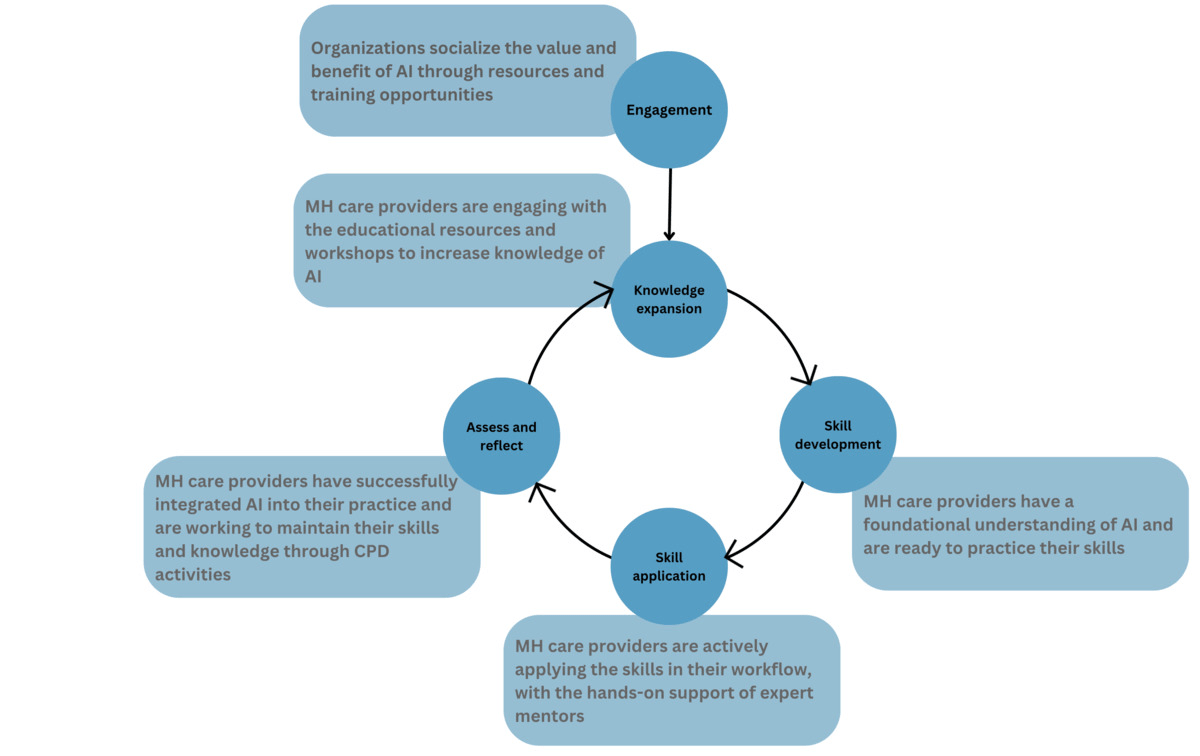
A framework for engaging mental health professionals in artificial intelligence (AI) based on the thematic analysis. CPD: continuing professional development; MH: mental health.

Initiatives such as the AI4People framework have been well established to guide the adoption of AI in society [26]. Although AI has been increasingly applied in health care, its applicability and efficacy in the context of mental health care remain an emerging topic [43]. Despite preliminary findings that show promise for AI technologies in this field [1,3,6-11,31], no guidance frameworks specific to the adoption and integration of AI exist for mental health professionals [22]. On the basis of our findings, which are in line with those of previous studies [44-46], we propose the following framework to address the AI adoption gap among mental health professionals (Figure 1).

First, it is important to engage mental health professionals in AI education initiatives (*Engagement*). For instance, organizational leaders should ensure that educational opportunities are made known to professionals and that they are readily accessible. In addition, major stakeholders, including AI developers, mental health professionals, clients, and families, should engage in a collaborative approach to co-design AI that is human-centered, reflecting the lived experiences of clients with mental illnesses [44,45].

Next, these initiatives must be undertaken by mental health professionals so that they can develop a foundational understanding of AI (eg, AI tools in practice, the efficacy of these tools, and equitable and inclusive AI; *Knowledge expansion*). In particular, although participants noted that learning technical aspects of AI, including how AI algorithms are developed, was not necessary, understanding who developed the tool and what kind of data were used, as well as seeing applications of AI within mental health care settings, would be valuable. Similar findings have been described in previous studies. For instance, a report from the National Academy of Medicine suggested that AI training for current and future care providers should emphasize the appropriate use and assessment of the tool [46]. Thus, future initiatives should address these gaps by first incorporating an overview of AI, including the process through which the tool was developed (eg, who developed it, the representativeness of the data used, and the validation method). As suggested by the extant literature and the findings of this study, this information could mitigate concerns regarding the validity and accuracy of the AI tool [22,45,47]. A comprehensive understanding of the equity, diversity, and inclusion of these tools may increase the willingness to adopt them in practice [45]. This education can be provided in a variety of ways, including integration into the undergraduate curriculum, workshops, and continuing education courses [44,45].

Once there is a basis of knowledge, it is important to develop the skills of mental health professionals so that they can use these tools in practice (*Skill development*). Participants noted that, in addition to being taught the broad scope of AI technologies, they would like to be taught how to use these technologies. Mental health professionals noted the importance of developing these skills in a hands-on manner (*Skill application*). Incorporating mentorship was noted as useful for ensuring the adoption of technology by professionals as having a mentor available to answer questions about a tool was seen as more helpful than didactic sessions. Real-life case scenarios of AI in mental health should be integrated into mentorship initiatives to clearly demonstrate the applicability of AI and its role in improving the quality of care.

Next, it is important to assess and reflect on the uptake of these technologies and determine professionals’ understanding and comfort with using AI in practice (*Assess and reflect*). During this period, organizations should collect feedback from major stakeholders to assess the functionality of educational activities and how they can be refined for future iterations. Finally, as AI continues to expand rapidly in the field, it is important that practitioners stay up-to-date by continuously engaging in educational initiatives to expand and practice their knowledge and skills.

### Limitations and Future Work

This study involved participants that identified as mental health professionals. Purposive sampling narrowed down the participant pool to those who belonged to our partner organizations, mostly in the province of Ontario, Canada. However, this may limit the perspectives of mental health professionals in other geographical locations. The research team is collecting other sources of data to broaden perspectives in subsequent studies. Although the participants were involved in mental health care, their level of education, exposure to and experience with AI, professional background, and interests varied. Finally, given that participants agreed to partake in this study, there is a possibility that they had more interest in the role of AI in mental health care than the average mental health professional. Therefore, a broad generalization of the findings should be carefully considered. However, the findings of this study may be interpretable for mental health professionals employed in the province of Ontario, Canada, and who have an interest in AI. Communication strategies are purported as a mechanism to address concerns regarding technology adoption, such as trust, value of the technology, and privacy issues [48]. Future research can focus on appropriate and effective communication to improve the adoption of AI in mental health settings.

### Conclusions

AI holds significant potential for the future of mental health care as it continues to evolve at a rapid pace. However, the adoption of AI tools in this sphere has experienced slow progress. As revealed in this needs assessment, education can play a transformative role in the attitudes and perspectives toward AI adoption and increase the understanding of AI among mental health professionals. The findings point to the importance of examining AI adoption at both the provider and organizational levels. As providers learn and adapt to these technologies, organizations must consider their role in supporting educational needs through training initiatives. To do so, appropriate funding, technological procurement, and organizational buy-in are necessary. Moving forward, future research should consider the role of organizational readiness in technological change and education in mental health care.

## Appendix

Multimedia Appendix 1 Interview guide.

## Abbreviations

AI: artificial intelligence
ASD: autism spectrum disorder

## Data Availability

The data sets generated and analyzed during this study are available from the corresponding author upon reasonable request.
