# Supplementary material for: The Adoption of AI in Mental Health Care–Perspectives From Mental Health Professionals: Qualitative Descriptive Study
Source: JMIR Form Res. 2023 Dec 7;7:e47847. doi: 10.2196/47847 (PMC10739240; doi:10.2196/47847)
Supplement: Multimedia Appendix 1 [file formative_v7i1e47847_app1.docx]

**Multimedia Appendix 1:**

**Accelerating the Adoption of AI in Mental Health Care Interview Guide**

**Practice Context:**

1. Please describe your current professional setting?
   1. Prompt: Where you work, what is your role?
   2. Prompt: Length of time in current role?

**Perception and familiarity with Digital, Data AI:**

1. What do you think of when you think of Artificial Intelligence (AI)?
   1. Prompt: what words or feelings come to mind?
2. How much do you feel you know about AI?
   1. Prompt: how familiar are you with AI?
   2. Prompt: how familiar are you with computer science?
   3. Prompt: how familiar are you with statistics?
   4. Prompt: what type of sources do you hear about AI from?
3. Please describe how you use digital and ehealth tools to support the delivery of mental health care?
4. How comfortable are you in using these tools to deliver care? Can you provide a positive example and one in which the outcome was different than expected?
5. How do you use data from your practice to improve your delivery of mental health care? Eg, patient reported outcomes, patient experience measures, data from an EMR/EHR

**Relevancy of AI for mental health professions**

1. In your opinion, how relevant is AI in the mental health care professions/practices?
   1. Prompt: Do you think there is a need for AI in mental health care?
   2. Prompt: How useful is AI in mental healthcare?
   3. Prompt: What role can AI play in mental healthcare delivery?
2. How comfortable would you be with integrating AI into your practice?
   1. Prompt: How likely would you use AI in your practice?
3. What would be any potential challenges you would have when using AI in your practice?
   1. Prompt: Is there anything that would make you feel hesitant to do so?
4. Would you say AI is prevalent in mental healthcare?
   1. Prompt: [If participant says yes] How is it prevalent? (e.g., do they hear about it a lot, where have they seen or heard about AI being used in mental health?)
   2. Prompt: [If participant says no] If not, why?
5. *If participant mentions organizational factors related to adoption of AI in mental health:*
   1. What are some organizational processes that either facilitate or hinder AI use in mental health care?
      1. Prompt: What role does the organization play in the process of AI adoption and implementation within mental health? (e.g., education, funding, collaboration)
      2. Prompt: [If participant describes hinderers] What are potential ways to mitigate this/change this?
      3. Prompt: At the organizational level, what should leaders do to overcome these barriers?
6. *If participant mentions barriers to AI use in mental health (e.g., lack of implementation):*
   1. What are some potential reasons for this to be happening?
   2. What are some ways to improve that?

**Education in AI**

1. What are some potential barriers for mental health professionals when learning about AI?
2. What are some potential positive aspects of learning about AI?
3. Are you aware of any education or training initiatives for using AI in mental healthcare delivery?
4. What information would you want to know about an AI technology that you could use in your practice?
5. When thinking about integrating AI into your practice, what would be some ways to help you feel more empowered doing so?
   1. Prompt: What aspects of your current practices would you want to remain unchanged?
   2. Prompt: What aspects of your current practices would you want to be changed?
6. Are you interested in attending an educational program or event relating to AI in mental health?
   1. Prompt: [If participant says yes] What led you to consider doing so?
   2. Prompt: [If participant says no] What are some reasons why you would not consider doing so?
